# Gut microbiota in hospitalized children with acute infective gastroenteritis caused by virus or bacteria in a regional Peruvian hospital

**DOI:** 10.7717/peerj.9964

**Published:** 2020-11-03

**Authors:** Andre Alonso Taco-Masias, Augusto R. Fernandez-Aristi, Angela Cornejo-Tapia, Miguel Angel Aguilar-Luis, Luis J. del Valle, Wilmer Silva-Caso, Victor Zavaleta-Gavidia, Pablo Weilg, Hernán Cornejo-Pacherres, Jorge Bazán-Mayra, Zully M. Puyen, Juana del Valle-Mendoza

**Affiliations:** 1 School of Medicine, Research and Innovation Centre of the Faculty of Health Sciences, Universidad Peruana de Ciencias Aplicadas, Lima, Peru; 2 Laboratorio de Biologia Molecular, Instituto de Investigación Nutricional, Lima, Peru; 3 Barcelona Research Center for Multiscale Science and Engineering, Departament d’Enginyeria Química, EEBE, Universitat Politècnica de Catalunya (UPC), Barcelona, Spain; 4 Dirección Regional de Salud de Cajamarca (DIRESA), Cajamarca, Peru; 5 Mercy One North Iowa Medical Center, Mason City, USA; 6 Centro Nacional de Salud Pública, Instituto Nacional de Salud, Lima, Peru

**Keywords:** Intestinal microbiota, Acute enteritis, Child, Bacterial infection, Viral infection, Gastrointestinal infection

## Abstract

**Background:**

Acute infective gastroenteritis (AIG) is a leading cause of mortality in children worldwide. In Peru, more than 40% of cases of AIG occurring in children under 5 years old. The disruption of the gut microbiota can increase risk for several health complications especially in patients with gastric infections caused by viruses or bacteria.

**Objective:**

The main objective of this study was to describe the prevalence of 13 representative bacteria from the gut microbiota (GM) in stools samples from children under 5 years of age with acute infective gastroenteritis.

**Results:**

The most commonly isolated bacteria from the GM were *Firmicutes* (63.2% 74/117) *Bacteriodetes* (62.4%; 73/117), *Lactobacillus* (59.8%; 70/117), *Prevotella* (57.2%; 67/117), *Proteobacterium* (53.8%; 63/117), regardless of the etiological agent responsible for the AIG. Interestingly, despite the high prevalence of *Firmicutes*, *Bacteroidetes*, *Lactobacillus* and *Prevotella* across all samples, a visible reduction of these agents was observed especially among patients with a single bacterial infection or even bacteria–bacteria coinfections when compared to viral etiologies. Patients with exclusive or mixed breastfeeding registered the highest amount of gut microbiota bacteria, in contrast to infants who received formula or were not breastfed.

## Introduction

The intestinal microbiota is made up of various types of microorganisms, including more than 4,000 different species of bacteria (Atarashi & Honda, 2011). These microorganisms evolved alongside the host so that they play a fundamental role in human physiology by modulating normal intestinal functions and participating in local and systemic immune responses (Chow et al., 2010). A better understanding of the interactions between the microbiota and the host’s immune system would help to understand the health and disease process that involves certain infectious and non-infectious pathologies such as inflammatory bowel disease (Lin & Zhang, 2017).

There are several factors that generate variations in the intestinal microbiota such as diet, geographical location, medication use, age, sex of the host, among others (Brunkwall & Orho-Melander, 2017; Pushpanathan et al., 2019). These variations can cause the loss of homeostasis between the intestinal microbiota and its surroundings, this is called dysbiosis, which is defined as the alteration in the composition and function of the microbiota. This condition has the ability to affect the permeability of the intestinal barrier, which causes inflammatory cascades, impaired mucosal integrity, among other harmful events at the intestinal level (Robles-Alonso & Guarner, 2013; Icaza-Chávez, 2013; Giglio, Burgos & Cavagnari, 2013; Devaraj, Hemarajata & Versalovic, 2013). In addition to this, several studies have described that alteration in the intestinal microbiota increases the risk of susceptibility to colonization with bacterial pathogens (Nelson et al., 2012).

Addressing the intestinal microbiota could represent a new approach to understand pathologies as frequent as infectious gastroenteritis, this because a significant reduction of the intestinal microbiota has been described in patients with gastroenteritis (Nelson et al., 2012; Chen et al., 2017).

In this context, acute gastroenteritis is characterized by the presence of diarrhea with or without vomiting and it is an important cause of morbidity and mortality in children. In general, the disease has a mild and self-limited clinical course, but in low-income countries, it represents a common cause of hospitalization (Pieścik-Lech et al., 2013; Guarino et al., 2012). It has been reported that acute gastroenteritis accounts for almost 2 million annual deaths in children under 5, and the highest mortality rates are concentrated in low-income countries (Elliot, 2007; O’Ryan et al., 2014). In Peru, a total of 119,417 episodes of acute gastroenteritis were reported in 2017, with more than 40% of cases in children under 5 years (Ordoñez, 2018).

Regarding gastroenteritis and its relationship with changes in the intestinal microbiota in patients under 5 years of age, the changes in the composition of the intestinal microbiota of patients with *Escherichia coli* (Shiga toxin –STEC) gastroenteritis producing have now been described, where be found a lower abundance of *Bifidobacterial* and *Clostridial* bacteria in infected infants (Gigliucci et al., 2018). Other studies in children with viral gastroenteritis describe microbiota changes in children with severe infectious gastroenteritis with greater abundance of *Prevotellaceae, Staphylococcaceae and Coriobacteriaceae* compared to healthy children (Chen et al., 2017).

Increasing evidence suggests that manipulation of the intestinal microbiota could treat or even prevent some intestinal diseases (Gigliucci et al., 2018; Sommer et al., 2017).

This is why the main objective of this study was to describe the prevalence of 13 bacteria representative of the intestinal microbiota in stool samples of children under 5 years of age with acute infective gastroenteritis (AIG).

## Material and Methods

### Patients and samples

A secondary analysis was performed on stools samples from a cross-sectional study in children under 5 years of age hospitalized due to an acute infective gastroenteritis (AIG) at *Hospital Docente Regional de Cajamarca* in rural Northern Peru (Cornejo-Tapia et al., 2017).

The original study enrolled 117 hospitalized children with AIG from January 2010 to December 2012 for etiological identification in stool samples of the following pathogens: Rotavirus, Adenovirus, Norovirus, *C. jejuni*, *C. coli*, *Shigella*, *Salmonella*, Enteroaggregative *E. coli* (EAEC) and Enteropathogenic *E. coli* (EPEC) (Cornejo-Tapia et al., 2017).

The diagnosis of AIG was defined as diarrhea lasting less than 14 days along with symptoms and signs such as fever, nausea, vomiting, and dehydration based on the guidelines’ criteria of the European Society for Pediatric Infectious Diseases. Nutritional status and dehydration status were based on the World Health Organization‘s criteria using Z-scores (weight for age).

PCR was used to detect 13 common gut microbiota (GM) bacterial genera including *Veillonella*, *Bacteroides*, *Bacteriodetes*, *Fusobacterium*, *Lactobacillus*, *Firmicutes*, *Actinobacterium*, *Bifidobacterium*, *Eubacterium*, *Prevotella*, *Enterococcus*, *Proteobacterium*, *Clostridium*.

### Ethics statement

This study was approved by the Ethics Committee from *Universidad Peruana de Ciencias Aplicadas* in Lima, Peru (Document No: FCS/291-12-17). The samples were obtained in the context of the epidemiological/syndromic surveillance program according to the health directives of the National Center for Epidemiology, Disease Control Prevention of the Ministry of Health of Peru. In this way, the collection of samples was exempt of informed consent.

### Samples

The samples from the original study were stored at −20 °C at the laboratory of the Research Center e Innovation for Health Sciences, Universidad Peruana de Ciencias Aplicadas (Lima, Peru).

### Nucleic acid extraction (DNA)

DNA extraction was performed from 200 µL of the fecal suspensions using a High Pure PCR template preparation kit (Roche Applied Science, Mannheim, Germany) performed according to the kit’s instructions.

### PCR amplification for detection gut microbiota

The 13 bacteria from the gut microbiota were amplified using nucleic acid extracted from fecal samples. Specific primers targeting different bacterial genera were used to characterize the fecal microbiota, these were previously described by Murri et al. (2013). Amplifications were initiated with an incubation at 95 °C for 2 min, followed by 40 cycles of 95 °C for 30 s, 58 °C for 30 s, and 72 °C for 30 s; with a final extension at 72 °C for 5 min. Amplified products were visualized on a 2.5% agarose gel containing 3 µg/mL of ethidium bromide in 1x Tris-borate buffer and photographed under ultraviolet illumination (UV Transilluminator KODAC LOGIC 1500, New Haven, USA). Amplified products were recovered from the gel, purified (SpinPrep™ Gel DNA Kit, San Diego, USA) and sent for commercial sequencing service (Macrogen, Seoul, Korea).

### Data analysis

Quantitative variables were described as frequencies and percentages for each group. The frequency distribution between groups was analyzed by *χ*^2^-Test of homogeneity using MiniTab v18.1 software (MiniTab Inc., USA). The authors could not get more data information such as type of delivery, daycare attendance, vaccines, diet and genetic factors; due to that this study in its objective original did not focus on these variables.

## Results

**Figure 1:**
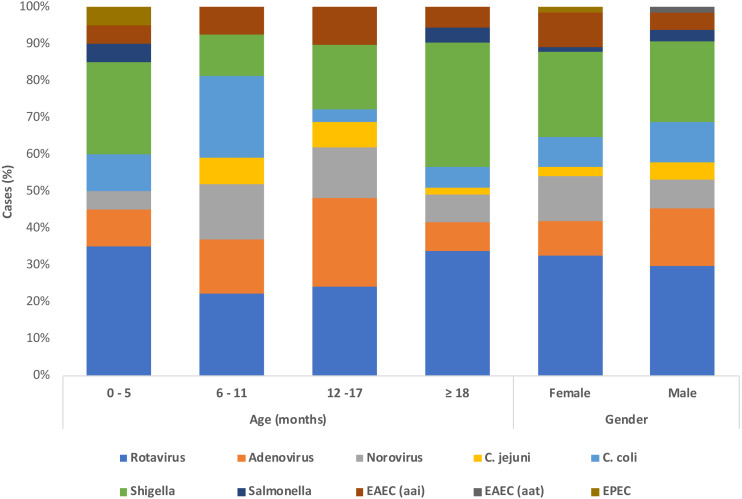
Demographics and etiological detection in patients with acute gastroenteritis.

A total of 117 samples from patients under 5 years old with AIG were analyzed via PCR to detect 13 bacteria from the gut microbiota (GM). In the population studied, infants younger than 12 months-old were the most common age group in 36.7% (43/117) followed by children older than 18 months old in 32.5% (38/117) and children 12–17 months in 23.9% (28/117), however there is a group of children of unknown age in 6.8% (8/117). Mixed breastfeeding was the most frequent practice in 39.3% (46/117), followed by exclusive breastfeeding in 29.1% (34/117). The attending physicians registered clinical symptoms at admission. Nausea and vomits were the most common complaint in 59% of patients (69/117), followed closely by fever in 54.7% (64/117). The distribution of each pathogen according to age, shows that the most predominant pathogens are rotaviruses followed by shigella, followed by adenovirus (Fig. 1).

**Figure 2:**
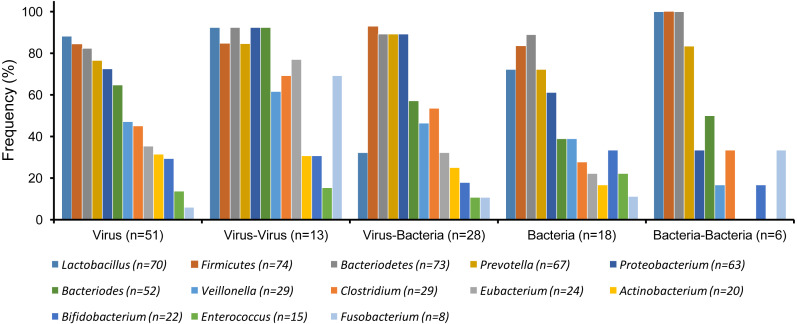
Gut microbiota detected in patients with single etiologies vs co-infections (*χ*^2^ test, *p* > 0.05).

**Table 1:** Gut microbiota detected in patients with viral and bacterial acute gastroenteritis (*χ*^**2**^**-Test,***p* > 0.05).

**Gut Microbiota**	**VIRUS**			**BACTERIA**						
	Rotavirus (*n* = 43)	Adenovirus (*n* = 17)	Norovirus (*n* = 14)	*C. jejuni* (*n* = 5)	*C. coli* (*n* = 13)	Shiguella (*n* = 31)	Salmonella (*n* = 3)	EAEC		EPEC
								aai (*n* = 10)	aat (*n* = 1)	eae (*n* = 1)
*Firmicutes* (*n* = 74)	37/43	16 /17	9/14	4/5	12/13	29/31	3/3	10/10	1/1	1/1
*Bacteriodetes* (*n* = 73)	37/43	15/17	11/14	4/5	11/13	29/31	3/3	10/10	1/1	1/1
*Lactobacillus* (*n* = 70)	39/43	14/17	13/14	4/5	13/13	26/31	3/3	9/10	1 /1	1/1
*Prevotella* (*n* = 67)	34/43	14/17	10/14	3 /5	10/13	26/31	3/3	10/10	1/1	1/1
*Proteobacterium* (*n* = 63)	35/43	12/17	11/14	4/5	13/13	24/31	3/3	8/10	1/1	1/1
*Bacteriodes* (*n* = 52)	28/43	13/17	12/14	2/5	6/13	17/31	2/3	7/10	0/1	0/1
*Clostridium* (*n* = 29)	23/43	6/17	9/14	1/5	2/13	1/31	2/3	7 /10	1/7	0/1
*Veillonella* (*n* = 29)	22/43	15/17	6/14	2/5	4/13	1/31	1/3	6/10	1/1	1/1
*Eubacterium* (*n* = 24)	16/43	6/17	8/14	1/5	2/13	5/31	1/3	4/10	1/1	0 /1
*Bifidobacterium* (*n* = 22)	12/43	2/17	5/14	2/5	3/13	4/31	0/3	3 /10	1/1	1/1
*Actinobacterium* (*n* = 20)	11/43	6/17	3/14	1/5	4/13	3/31	0/3	1/10	1/1	0/1
*Enterococcus* (*n* = 15)	5 /43	4/17	2/14	0/5	3/13	2/31	1/3	0/10	0/1	1/1
*Fusobacterium* (*n* = 8)	4/43	0 /17	1 /14	0/5	1/13	4/31	1/3	1/10	0/1	0/1

In general, the distribution of GM for different types of gastrointestinal infection (e.g., virus, bacteria or their coinfections) were similar or showed no significant differences between them (*χ*^2^-Test, *p* > 0.05) (Fig. 2). However, the most commonly isolated bacteria from the GM were *Firmicutes* (74/117), *Bacteriodetes* (73/117), *Lactobacillus* (70/117), *Prevotella* (67/117), *Proteobacterium* (63/117). These five bacteria were also the most frequently isolated in all samples regardless of their etiological agents isolated (Table 1). Interestingly, despite the high prevalence of *Firmicutes*, *Bacteroidetes*, *Lactobacillus*, and *Prevotella* across all samples, a visible reduction of these agents was observed especially among patients with a single bacterial infection or even bacteria –bacteria coinfections when compared to viral etiologies (Fig. 2) (Table S1).

**Figure 3:**
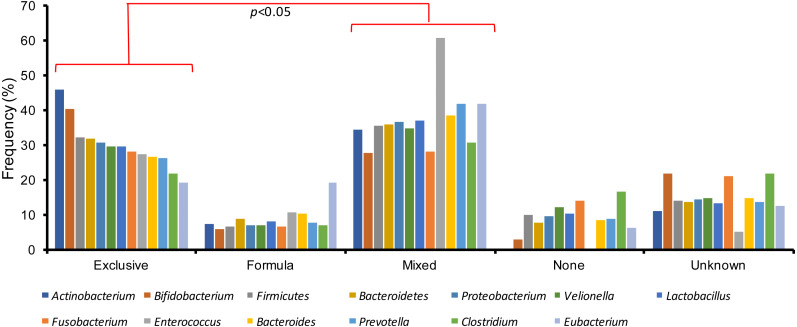
Gut microbiota variations among the different types of breastfeeding in children with acute gastroenteritis.

Finally, gut microbiota variations were also studied among the several types of breastfeeding practices in the children with an acute gastroenteritis. Patients with exclusive or mixed breastfeeding registered the highest amount of gut microbiota bacteria, in contrast to infants who received formula or were not breastfed (*χ*^2^-Test, *p* < 0.05) (Fig. 3). In addition, exclusive and mixed breastfeeding were significantly different (*χ*^2^-Test, *p* < 0.05).

## Discussion

The gut microbiota (GM) is a complex community of microorganisms inhabit in the human gastrointestinal tract. This collection of bacteria, archaea, and eukarya offer benefits to the host strengthening gut integrity, protecting against potential pathogens and regulating host immunity (Van den Elsen et al., 2017; Goulet, 2015; Petrof et al., 2013; Munoz et al., 2013; Human Microbiome Project C, 2012; Martz et al., 2014; Tremaroli & Bäckhed, 2012; Schippa & Pia-Conte, 2014). However, alterations in the microbiota composition (dysbiosis) can result in the disruption of these protective mechanisms increasing their risk for infections (Van den Elsen et al., 2017; Goulet, 2015; Thursby & Juge, 2017).

The most common cause of AIG in children is viruses in approximately 70% of cases, followed by bacteria in 10–20% of patients (Elliot, 2007; Chen et al., 2006). Among the viral etiologies, Rotavirus is the most frequent pathogen with different viral strains varying by season and geographies and affecting primarily children under 5 years old (Elliot, 2007; Chen et al., 2006; Shah et al., 2017). However, in developing countries, pathogenic bacteria can be the leading cause of AIG with *E. coli* being the most commonly isolated etiology followed by *Shigella flexneri* (Elliot, 2007; Dedwal, Pol & Bharadwaj, 2016; Kotloff et al., 2013). In this study, a viral etiology was isolated in 63.2% of samples and bacteria were detected in 54.7%. Rotavirus was the most common etiology detected in 36.8% (43/117) of samples, followed by *Shigella* in 26.5% (31/117).

Undernutrition in children is associated with a reduced microbiota diversity, characterized by a low prevalence of *Bacteroidetes* and a high number of *Proteobacteria*. This susceptible population has a higher infection risk for *Klebsiella* and *Escherichia* (Pekmez, Dragsted & Brahe, 2018; Monira et al., 2011; Goday, 0000; Chiejina & Samant, 2018). Moreover, a study in India found that infections due to *Escherichia*, *Streptococcus*, *Shigella*, *Enterobacter* and *Veillonella* generate are more frequent in children with a malnutrition (Pekmez, Dragsted & Brahe, 2018). In this study, only 14.5% (17/117) of patients had chronic malnutrition as a risk factor for acute gastroenteritis and only 4 cases of Shigellosis and 3 cases of EAEC were observed. Moreover, *Proteobacterium* were less common among patient with a bacterial AIG.

Studies have shown that breastfeeding provides prebiotics and bacterial communities that stabilize the GM during the first year of life, including *Bifidobacterium*, *Lactobacillus* and *Bacteriodetes* (Yu et al., 2013; Bäckhed et al., 2015). This beneficial effect has been reported in breastfed and mixed-fed infants who acquire a greater amount of bacteria from the human milk compared to their counterparts (Monira et al., 2011). The most patients had mixed feeding (39.3%), followed by those who received exclusive breastfeeding (29.1%). The GM bacteria in those feeding patterns that included human milk presented a higher frequency of detection especially in the *Bifidobacterium, Actinobacterium and Enteroccus* genera. This finding further supports the study of Bäckhed et al. (2015) who showed that the sole only presence of human milk predisposes to greater GM diversity, compared with the population that was not breastfed. However, we must stand out there are some bacteria that need being supported by human milk or formula such as *Enterococcus* and *Actinobacteirum*, that were undetectable in people that received none, as we can note in this study.

The most prevalent microbiota bacteria were *Firmicutes* (63.2%), *Bacteriodetes* (62.4%), *Lactobacillus* (59.8%), *Prevotella* (57.3%), *Proteobacterium* (53.8%) and *Bacteroides* (44.4%). On the contrary, *Clostridium* (24.8%), *Veillonella* (24.8%), *Eubacterium* (20.5%), *Bifidobacterium* (18.8%), *Actinobacterium* (17.1%), *Enterococcus* (12.8%) and *Fusobacterium* (6.8%) were the least common detected. These last three can guide us that the simple presence of any etiological agent, whether viral or bacterial, it can be disruptive for these GM bacteria. That is why those who can generate any alteration in their homeostasis are principally the cytotoxic bacteria such as *Shigella* and *E. coli* species. Such is so, the low detection of *Enterococcus, Bifidobacterium* and *Fusobacterium* is due to the presence of *Shigella flexneri* and EAEC. However, we shall state that some viral aetiologies, such as double stranded linear DNA virus, can be dangerous for Enterococcus due to these virus, specially *Adenovirus*, can disturb the intestinal pH generating an acid pH leading to death of *Enterococcus,* that lives in alkaline pH (Rexroad et al., 2016). As stated above, the decrease of GM communities are correlated with chronic disease due to the persistent inflammatory state created by dysbiosis (Kowalska-Duplaga et al., 2019). Many of these ones are autoimmune diseases and they are linked to this trend such as Crohn’s disease in which the pivot GM finding is the decreased *Bifidobacterium* and *Fusobacterium* (Pittayanon et al., 2019). In contrast to ulcerative colitis that has the same pattern of GM, but with a more predominant decrease in *Fusobacterium* (Wang et al., 2018). On the other hand, there are other contexts, such as Henoch-Schönlein purpura where the keystone is the detection of *Enterococcus* who play a role in the pro-inflammatory state described above (Gosh et al., 2014). Although this study has used a qualitative assay, we have observed the absence of these GM communities. We are also able to hypothesis that the lack of those specific bacterial communities exerts the pro-inflammatory states in chronic diseases and may play a role as protective GM bacteria. However, it is difficult to speculate what these frequencies implies in these patients since this is a new research field. Nonetheless, based on currently published studies on these bacteria microbiota different hypotheses can be proposed.

The human GM contains at least 1000 different species of known bacteria, protective mechanisms against AIG have been described in only a few of them. For example, *Bacteroides*, which is the most predominant anaerobic bacteria in the human gastrointestinal tract (HGIT), provide sugar molecules through carbohydrate fermentation which are important in the activation of T-cell-dependent immune response, and the expression of protective proteins from the Paneth cell (defensins and Ang4) (Wexler, 2007). *Proteobacterium*, play an important role in the transition from the neonatal to adult microbiota, favoring colonization by obligate anaerobes; which later are replaced by *Firmicutes* and *Bacteroidetes*. These bacteria were commonly detected in these patients, with no significant difference in their prevalence across the different etiologies isolated. However, an increased number of *Firmicutes* and *Bacteroidetes* in the gut can be used as an indicator of dysbiosis (Shin, Whon & Bae, 2015; Ramakrishna, 2013).

Other important bacteria are *Lactobacillus*, which are a major probiotic factor for other bacteria, as well as a modifier of the cell surface glycan, enhancing the action of *Bacteroidetes* (Schippa & Pia-Conte, 2014). Additionally, *Lactobacillus* serves as a microbiota stabilizer between other communities exchanging DNA traits and other soluble factors (Szajewska et al., 2013). *Lactobacillus* was common in these patients and some strains, such as *Lactobacillus* GG may reduce the duration of diarrhea in AIG (Dubin & Pamer, 2014).

*Enterococcus* contributes to colonic homeostasis through PPAR*γ*1-induced IL-10 and TGF-ßexpression and can reduce the severity of infectious diarrhea in children (Odamaki et al., 2016). Furthermore, *Enterococcus* is used as a probiotic, as well as part of the fecal microbiota transplant along with *Lactobacillus casei* (Odamaki et al., 2016). In patients’ samples, *Enterococcus* was less commonly isolated, especially in the patients with shigellosis in which an inflammatory destruction of the intestinal barrier is usually observed.

*Bifidobacterium* its main role is to down-regulate pro-inflammatory responses in the gut epithelium and *Clostridium* spp. that provides substrates from products of fermentation acting as an impressive anti-inflammatory agent by avoiding the activation of the transcription factor, NF-*κ*B (Lopetuso et al., 2013; Sun & Kato, 2016). Mainly, both bacteria were not detected in this study. *Veillonella* and *Fusobacterium* were expected to be less commonly isolated as both have a preference for oral cavity, working as a bridge organism between early and late colonizers (Sun & Kato, 2016).

This study is among the first ones to describe the different prevalence of the most important bacteria from the GM in patients with multiple etiologies of AIG. However, as we have discussed previously it is difficult to hypothesize what these frequencies implies in the patient due to the study limitations.

The first study limitation was the lack of normal range values to determine if the number of bacteria from the GM we isolated are decreased or augmented. Nonetheless, we tried to compare these findings with GM bacteria known to be more predominant in different regions of the gastrointestinal tract. Second, since the only patient with AIG were studied it is not possible to compare this prevalence with a normal equivalent population. Finally, although interesting findings such as the visible reduction of GM bacteria among patients with a bacterial infection or the higher number of GM in a patient receiving exclusive or mixed breastfeeding were worth mentioning; to imply a cause–effect relationship is not possible.

## Conclusion

In conclusion, despite the etiology of the infection, the most frequent bacteria identified were *Firmicutes*, *Bacteroidetes*, *Lactobacillus*, *Prevotella*, and *Proteobacterium*. However, there are some important bacteria which has a crucial role maintaining the integrity of the GM and the enteric epithelium among all described GM bacteria, such as *Bacteroidetes*, *Enterococcus*, *Bifidobacterium* and *Actinobacterium*, whose the main role is stabilize the GM. It is very important to note that the microorganisms most affected are the ones who promote the homeostasis and the continuity of the intestinal microbiome. In addition, we have to take into account the *Actinobacterium* and the *Bifidobacterium* whose main role falls in the modulation of gut permeability and the main axis, which play a crucial role when there is a pro-inflammatory state, either an infection or a systemic disease, promoting a protective barrier in the mucosa, and stabilizing the GM along with *Bacteroidetes*.

These data guide us that every GM bacteria has an important role to maintain the homeostasis. For all of above, we can conclude that the current treatment regimens must focus on a symbiotic therapy, which content more gut microbiota bacteria instead of the single regimen, such as *Lactobacillus* regimens, which are the most used ones in the AIGs, since the bacteria of the intestinal microbiota are distributed in a same way in viral, bacterial gastroenteritis or in various coinfections.

##  Supplemental Information

10.7717/peerj.9964/supp-1 Supplemental Information 1 Gut Microbiota detected in patients with co-infections in acute gastroenteritis (*c*^2^-Test, *p*> 0.05).

## Additional Information and Declarations

### Data Availability

The following information was supplied regarding data availability: The raw data is available at Figshare: Del Valle Mendoza, Juana; Taco-Masias, André; Fernández-Aristi, Rafael; Tapia, Angela Cornejo; Weilg, Pablo; Zavaleta-Gavidia, Victor; et al. (2020): Dataset Microbiota AG y2020m03. figshare. Dataset. https://doi.org/10.6084/m9.figshare.12009492.v1.
