# Supplementary material for: Gut microbiota in hospitalized children with acute infective gastroenteritis caused by virus or bacteria in a regional Peruvian hospital
Source: PeerJ. 2020 Nov 3;8:e9964. doi: 10.7717/peerj.9964 (PMC7646295; doi:10.7717/peerj.9964)
Supplement: Supplemental Information 1 [file peerj-08-9964-s001.pdf]

Gut Microbiota	Virus (n=51)	Virus-Virus (n=13)	Virus- Bacteria (n=28)	Bacteria (n=18)	Bacteria-Bacteria (n=6)
Firmicutes (n=74)	43/51	11/13	26/28	15/18	6/6
Bacteriodetes (n=73)	42/51	12/13	25/28	16/18	6/6
Lactobacillus (n=70)	45/51	12/13	9/28	13/18	6/6
Prevotella (n=67)	39/51	11/13	25/28	13/18	5/6
Proteobacterium (n=63)	37/51	12/13	25/28	11/18	2/6
Bacteriodes (n=52)	33/51	12/13	16/28	7/18	3/6
Clostridium (n=29)	23/51	9/13	15/28	5/18	2/6
Veillonella (n=29)	24/51	8/13	13/28	7/18	1/6
Eubacterium (n=24)	18/51	10/13	9/28	4/18	0/6
Bifidobacterium (n=22)	15/51	4/13	5/28	6/18	1/6
Actinobacterium (n=20)	16/51	4/13	7/28	3/18	0/6
Enterococcus (n=15)	7/51	2/13	3/28	4/18	0/6
Fusobacterium (n=8)	3/51	9/13	3/28	2/18	2/6

Eliminado: **Table S1. Gut Microbiota detected in patients with co-infections in acute gastroenteritis (χ^2-Test, p > 0.05).**
